# Testicular lymphoma of 63 patients: a Chinese retrospective, real-world study

**DOI:** 10.3389/fonc.2025.1652944

**Published:** 2025-10-30

**Authors:** Honghan Qiao, Sijun Zhang, Yukai Duan, Renjie Hua, Feiyang Zong, Mingzhi Zhang, Xudong Zhang

**Affiliations:** Department of Oncology, the First Affiliated Hospital of Zhengzhou University, Zhengzhou, China

**Keywords:** testicular lymphoma, diffuse large B-cell lymphoma, central nervous system, survival, real-world study

## Abstract

**Objective:**

To suggest the difference between primary and secondary testicular lymphoma, and to manifest the clinical characteristics, treatment modalities and prognostic factors of primary testicular lymphoma.

**Method:**

This study included all lymphoma patients with testicular involvement treated at our institution between October 2012 and May 2024. We retrospectively collected data on their clinical characteristics, treatment approaches, and outcomes for further analysis.

**Result:**

A total of 50 primary testicular lymphoma (PTL) patients and 13 secondary testicular lymphoma (STL) patients were enrolled, with diffuse large B-cell lymphoma (DLBCL) being the most common subtype. After a median follow-up of 36.0 months (range: 1.1–117.5), the median progression-free survival (PFS) was 105.9 months for PTL patients and 16.8 months for STL patients. The median overall survival (OS) was 106 months for PTL and 23.8 months for STL. Among the 46 primary DLBCL (PT-DLBCL) cases, half received central nervous system (CNS) prophylaxis, and 7 patients (15.2%) experienced CNS relapse. Patients who received maintenance therapy after orchiectomy and first-line treatment exhibited prolonged PFS. Radiotherapy was associated with improved PFS, while the double-expressor phenotype was linked to poorer OS.

**Conclusion:**

PTL suggested distinct histopathological features, clinical responses, and survival outcomes compared to STL. A combined treatment strategy involving orchiectomy followed by chemotherapy, consolidation therapy, and radiotherapy is recommended for PT-DLBCL. As intrathecal methotrexate did not significantly reduce CNS recurrence, alternative prophylactic strategies should be explored for high-risk patients.

## 1 Introduction

The testes are regarded as immune-privileged sites, protected by the blood-testicular barrier and immunomodulatory mechanisms. Lymphomas arising in these immune sanctuaries represent a rare subtype of non-Hodgkin lymphoma (NHL), typically associated with aggressive clinical behavior and poor prognosis. These lymphomas can be broadly categorized as either primary testicular lymphoma (PTL) or secondary testicular lymphoma (STL).

PTL is defined by the presence of testicular masses as the initial or predominant manifestation, without obvious involvement of other extranodal organs. In contrast, STL is caused by the spread of extara-testicular lymphoma. Clinically, differentiating PTL from STL can be challenging, particularly when testicular involvement is the presenting symptom. Accurate clinical staging is also crucial for determining the appropriate treatment. PTL accounts for 1% to 2% of all NHL cases and 3% to 9% of testicular malignancies. It is the most common testicular tumor in men over 60 and is associated with a high risk of contralateral testicular and central nervous system (CNS) relapse (1). The majority of PTL cases are diffuse large B-cell lymphoma (DLBCL), while STL displays more varied histopathological types.

Although the incidence of PTL is gradually increasing in China, the overall occurrence of testicular lymphoma remains significantly lower in Asian populations compared to Western cohorts. This discrepancy has resulted in a scarcity of real-world clinical data from Chinese populations. Furthermore, despite the consensus on standard PTL treatment, optimal therapeutic approaches following orchiectomy remain a subject of debate. Novel agents, such as PD-1 and Bruton’s tyrosine kinase inhibitors (BTK-i), have shown promise. In light of these issues, we conducted this retrospective study, collecting real-world data from PTL and STL patients at our center. Our aim was to compare clinical and pathological characteristics, assess the effectiveness of various treatment regimens, and evaluate survival outcomes.

## 2 Materials and methods

### 2.1 Study design and patient selection

**Figure 1:**
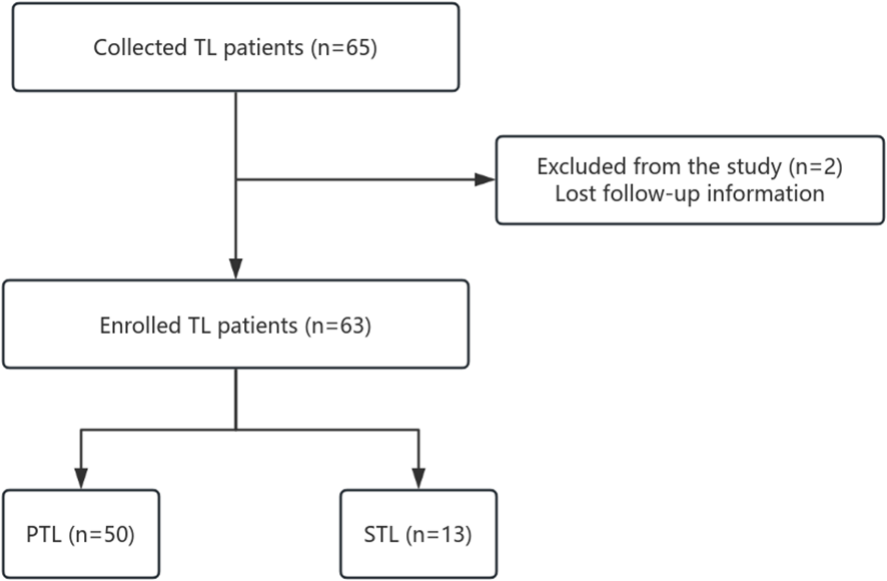
Procedures of patients selection.

We retrospectively collected data from 63 lymphoma patients with testicular involvement treated at the First Affiliated Hospital of Zhengzhou University between October 2012 and May 2024. Patients were categorized into two groups: primary testicular lymphoma (PTL) and secondary testicular lymphoma (STL). Procedures of patient selection are displayed in **Figure 1**. The classification was based on whether the testicular mass was the primary or predominant symptom and whether there was significant involvement of other extranodal organs. PTL was confirmed through testicular pathology, while testicular involvement in STL cases was evaluated using either pathology or positron emission computed tomography (PET/CT). Disease staging was conducted according to the Ann Arbor staging system. Immunohistochemistry was performed for Bcl-2 and c-Myc expression, while fluorescence *in situ* hybridization (FISH) was used to identify double-expressor types as well as double-hit/triple-hit DLBCL. The cell of origin (COO) of DLBCL was determined using the HANS algorithm, based on the expression of CD10, Bcl-6, and MUM-1. Clinical characteristics such as age, Ann Arbor stage, extranodal involvement, treatment regimens, and disease response were collected for further analysis. This study was approved by the Ethics Committee of the First Affiliated Hospital of Zhengzhou University (2022-KY-0869-001). All the processes conformed to the Declaration of Helsinki. All patients have signed written informed consents before treatment.

### 2.2 Treatment modalities and assessment of effectiveness

For PTL patients, orchiectomy was followed by 4 to 6 cycles of chemotherapy, CNS prophylaxis, and radiotherapy (RT). Prophylactic contralateral testicular RT or nodal RT after first-line treatment was administered with a total dose ranging from 20 to 45 grays. CNS prophylaxis was primarily carried out through intrathecal injections of methotrexate (MTX, 12–15 mg), cytarabine (AraC, 50 mg), and dexamethasone (DXM, 5 mg). Treatment response was evaluated using computed tomography (CT) or positron emission tomography/computed tomography (PET/CT). Short-term effectiveness was classified as complete response (CR), partial response (PR), stable disease (SD), or progressive disease (PD). The overall response rate (ORR) was defined as the percentage of patients achieving CR or PR. Overall survival (OS) was measured from the date of diagnosis to death or last observation from any cause, while progression-free survival (PFS) was defined as the time from diagnosis to the first relapse, progression, death, or last follow-up.

### 2.3 Statistical analysis

Descriptive statistics were expressed as percentages and medians. Treatment effectiveness was compared using the chi-squared test. Survival outcomes, including overall survival (OS) and progression-free survival (PFS), were estimated using the Kaplan–Meier method. Differences between survival curves were compared using the log-rank test. Univariate Cox proportional hazards regression models were applied to identify potential prognostic factors. Variables assessed included age at diagnosis, Ann Arbor stage, COO, laterality, IPI score, immunophenotype, first-line therapy, radiotherapy, maintenance therapy and intrathecal CNS prophylaxis. Variables with a p-value <0.05 in univariate analysis were further included in the multivariate Cox model. All statistical analyses were conducted with SPSS software version 26.0 and GraphPad Prism version 8. A p-value of <0.05 was considered statistically significant.

## 3 Results

### 3.1 Baseline characteristics, clinical response, and survival outcomes between PTL and STL patients

A total of 63 patients with testicular involvement were included in the study, consisting of 50 PTL and 13 STL patients. Comparisons between their clinical characteristics and their treatment options are depicted in **Table 1**. The median age (range) for PTL patients was 64 years (39–88), and for STL patients, it was 53 years (29–77). Among them, 56.0% of PTL patients and 30.8% of STL patients were over 60 years of age. All PTL cases and 6 STL cases were confirmed via testicular pathology, while the remaining 7 STL patients were assessed using FDG-PET/CT, with a mean testicular maximum standardized uptake value (SUVmax) of 12.3 (range: 5.0–21.1). The majority of patients were diagnosed with DLBCL, accounting for 92% of PTL and 53.8% of STL cases, with a statistically significant difference in pathology between the two groups (p < 0.001). Of the PTL patients, 52% (n = 26) presented with limited-stage lymphoma (stage I E and II E), while all STL patients were diagnosed at an advanced stage. Two PTL and one STL patient had a history of testicular injury, and four PTL and two STL patients had undergone testicular surgery, potentially linked to the development of testicular lymphoma. Most patients had unilateral disease, observed in 86% of PTL and 76.9% of STL patients. Bone marrow involvement was noted in four PTL (8%) and three STL (23.1%) patients, while CNS involvement occurred in seven PTL (14%) and four STL (30.8%) patients. During follow-up, 22 patients died, including four PTL and nine STL patients, with a significant difference in mortality between the groups (p = 0.01).

**Table 1:** Baseline characteristics and treatment modalities.

Characteristics, n (%)	PTL (n=50)	STL (n=13)
Age		
Median (range) age, years >60 years ≤60 years	64 (39-88) 28 (56.0%) 22 (44.0%)	53 (29-77) 4 (30.8%) 9 (69.2%)
Disease diagnosis		
DLBCL NKTCL MCL PBL TFH	46 (92.0%) 1 (2.0%) 1 (2.0%) 2 (4.0%) 0 (0.0%)	7 (53.8%) 5 (38.5%) 0 (0.0%) 0 (0.0%) 1 (7.7%)
Ann Arbor stage		
IE IIE III IV	13 (26.0%) 13 (26.0%) 2 (4.0%) 22 (44.0%)	0 (0.0%) 0 (0.0%) 0 (0.0%) 13 (100%)
Location		
Left Right Bilateral	19 (38.0%) 24 (48.0%) 7 (14.0%)	3 (23.1%) 7 (53.8%) 3 (23.1%)
Extranodal involvement		
Bone marrow involvement CNS involvement Kidney Subcutaneous tissue Adrenal gland Pancreas Parotid gland Ureter Bone Nasal cavity	4 (8.0%) 7 (14.0%) 3 (6.0%) 2 (4.0%) 2 (4.0%) 1 (2.0%) 1 (2.0%) 1 (2.0%) 1 (2.0%) 0 (0.0%)	3 (23.1%) 4 (30.8%) 1 (7.7%) 0 (0.0%) 0 (0.0%) 0 (0.0%) 1 (7.7%) 0 (0.0%) 2 (15.4%) 2 (15.4%)
Survival		
Alive Death of any cause	37 (74.0%) 13 (26.0%)	4 (30.8%) 9 (69.2%)
Orchiectomy		
Yes No	48 (96.0%) 2 (4.0%)	6 (46.2%) 7 (53.8%)
First-line therapy		
R-CHOP-like regimens^*^ CHOP^*^ R-EPOCH^*^ DDGP^*^ Gemox^*^ Others	33 (66.0%) 4 (8.0%) 4 (8.0%) 1 (2.0%) 0 (0.0%) 6 (12.0%)	7 (53.9%) 1 (7.7%) 0 (0.0%) 3 (23.1%) 1 (7.7%) 1 (7.7%)
Radiotherapy		
Yes No	29 (58.0%) 21 (42.0%)	6 (46.2%) 7 (53.8%)
CNS prophylaxis		
Yes No	31 (62.0%) 19 (38.0%)	6 (46.2%) 7 (53.8%)
HSCT		
Yes No	1 (2.0%) 49 (98.0%)	1 (7.7%) 12 (92.3%)
CAR-T		
Yes No	0 (0.0%) 50 (100.0%)	2 (15.4%) 11 (84.6%)

^*^R-CHOP: rituximab, cyclophosphamide, doxorubicin, vincristine, prednisone;

^*^CHOP: cyclophosphamide, doxorubicin, vincristine, prednisone;

^*^R-EPOCH: rituximab, etoposide, prednisone, vincristine, cyclophosphamide, doxorubicin;

^*^DDGP: dexamethasone, cisplatin, gemcitabine, pegaspargase;

^*^Gemox: gemcitabine, and oxaliplatin.

At a median follow-up of 36.0 months (range: 1.1–117.5), treatment response and survival outcomes were evaluated for all patients. The overall response rate (ORR) was 61.9%, with 31 patients achieving a complete response (CR) and eight patients achieving a partial response (PR). The total CR rate was 49.2% (**Table 2**). Among PTL patients, the ORR was 70.0%, with 58.0% achieving a CR, while in STL patients, the ORR was 30.8%, with 15.4% achieving a CR, representing a significant difference between the two groups (p = 0.023).

**Table 2:** Clinical response in PTL and STL patients.

Effectiveness,n (%)	Total,63	PTL,50	STL,13	P value
ORR	39 (61.9%)	35 (70.0%)	4 (30.8%)	0.023
CR	31 (49.2%)	29 (58.0%)	2 (15.4%)	
PR	8 (12.7%)	6 (12.0%)	2 (15.4%)	
SD	6 (9.5%)	5 (10.0%)	1 (7.7%)	
PD	18 (28.6%)	10 (20.0%)	8 (61.5%)	

**Figure 2:**
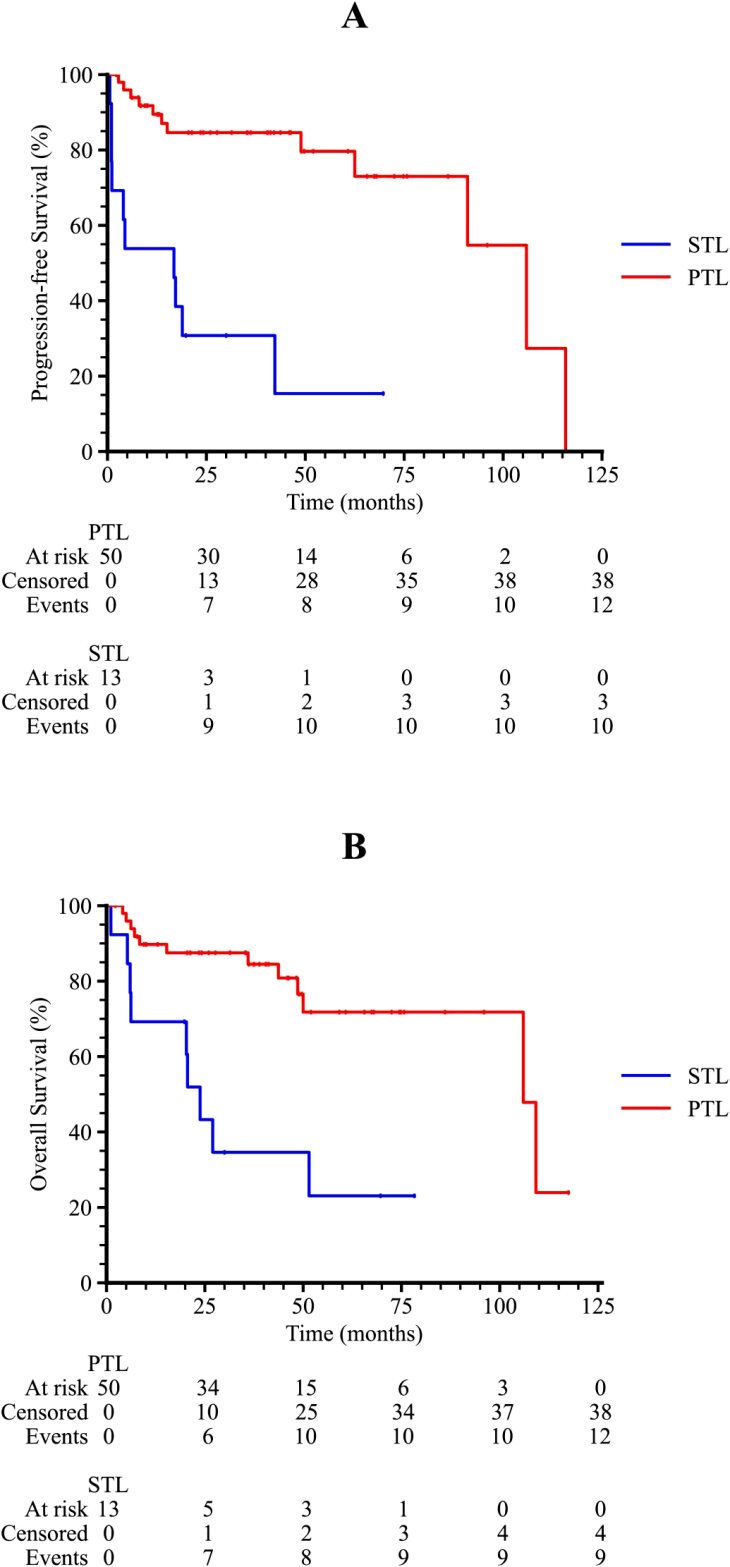
Kaplan-Meier survival curves of TL patients. **(A)** The progression-free survival curve among PTL and STL patients. **(B)** The overall survival curve among PTL and STL patients.

Kaplan-Meier survival analysis showed that both progression-free survival (PFS) and overall survival (OS) were significantly better in PTL patients compared to STL patients (p < 0.001 and p = 0.001, respectively, **Figure 2**). The 3-year PFS and OS rates for PTL patients were 84.6% and 81.9%, respectively, whereas for STL patients, the 3-year PFS was 30.8% and the 3-year OS was 34.6%. The median PFS for PTL and STL patients was 105.9 months and 16.8 months, respectively, and the median OS was 106 months and 23.8 months, respectively.

### 3.2 Primary testicular DLBCL

#### 3.2.1 Clinical features and treatment regimens of primary testicular DLBCL

A total of 46 primary testicular DLBCL (PT-DLBCL) patients were enrolled in the study, representing the majority of PTL cases. The clinical characteristics and treatment modalities of these patients are summarized in **Table 3**. The most commonly reported primary symptom was unilateral painless swelling (50.0%), followed by testicular pain (26.1%), a combination of swelling with pain (17.4%), and the presence of a testicular mass (17.4%). Fourteen out of 46 patients (30.4%) had relapsed or refractory (R/R) disease, and 78.2% of cases were of the non-GCB subtype. MYD88 gene testing was performed on five patients, with four testing positive for the MYD88 mutation. All patients were diagnosed with DLBCL through pathological examination, with 44 out of 46 (95.7%) undergoing orchiectomy, while two patients were confirmed through testicular puncture biopsy without receiving any orchiectomy.

**Table 3:** Clinical features of primary testicular DLBCL.

Clinical features	N (%)
Primary symptoms	46 (100%)
Unilateral painless swelling Testicular pain Swelling of testis with pain Testicular mass Fever Abdominal pain	23 (50.0%) 12 (26.1%) 8 (17.4%) 8 (17.4%) 4 (8.7%) 2 (4.3%)
Disease status	
Relapse Refractory	2 (4.3%) 12 (26.1%)
IPI score	
0-1 2-3 4-5	19 (41.3%) 19 (41.3%) 8 (17.4%)
CNS-IPI score	
0-1 2-3 4-6	19 (41.3%) 17 (37.0%) 10 (21.7%)
Cell of origin	
Non-GCB subtype GCB subtype	36 (78.2%) 10 (21.7%)
Immunophenotype	
Double-expressor phenotype	10 (21.7%)
Double/triple-hit phenotype MYD88	4 (8.7%) 4 (8.7%)

Detailed information on treatment regimens is summarized in **Table 4**. Among all patients, the majority received R-CHOP-like regimens as first-line treatment. These included R-CHOP, Rituximab with cyclophosphamide, pegylated liposomal doxorubicin, vincristine, and prednisone (R-CDOP); zanubrutinib with R-CHOP (ZR-CHOP); and lenalidomide with R-CHOP (R2-CHOP). Two patients did not receive chemotherapy after orchiectomy. After a median of four cycles of frontline treatment, 11 patients received Rituximab (R) monotherapy as consolidation therapy. Several second-line and maintenance regimens were used in relapsed or high-risk cases, including lenalidomide, thalidomide, and R-Gemox. Additionally, 28 out of 46 patients underwent radiotherapy following chemotherapy, and CNS prophylaxis was given to 28 patients (60.9%), including 27 patients with intrathecal methotrexate and 1 with intravenous methotrexate.

**Table 4:** Summary of treatment regimens in patients with primary testicular DLBCL.

Treatment category	Regimen or approach	N (%)
Orchiectomy	Yes No	44 (95.7%) 2 (4.3%)
First-line therapy	R-CHOP-like regimens CHOP R-EPOCH NO Others	32 (69.6%) 4 (8.7%) 3 (6.5%) 2 (4.3%) 5 (10.9%)
Number of first-line treatment cycles	Median (range)	6 (0-10)
R as consolidation therapy	Yes No	11 (23.9%) 35 (76.1%)
Second-line therapy	R-Gemox^*^ BR^*^ Gemox R-GDP^*^	3 (6.5%) 2 (4.3%) 1 (2.1%) 1 (2.1%)
Maintenance therapy	Lenalidomide Thalidomide Pomalidomide BTK-i Rituximab Cyclophosphamide No	7 (15.2%) 2 (4.3%) 1 (2.1%) 7 (15.2%) 1 (2.1%) 1 (2.1%) 27 (58.7%)
CNS prophylaxis	Intrathecal (IT) Number intrathecal injections, median (range) Intravenous (IV) methotrexate No	27 (58.7%) 4 (0-7) 1 (2.1%) 18 (39.1%)
Radiotherapy	Yes No	28 (60.9%) 18 (39.1%)

^*^R-Gemox: rituximab, gemcitabine, and oxaliplatin;

^*^BR: bendamustine and rituximab;

^*^GDP: gemcitabine, dexamethasone, cisplatin; Gemox: gemcitabine and oxaliplatin

#### 3.2.2 Contralateral testis and CNS recurrence in primary testicular DLBCL

Among the 46 PT-DLBCL patients, 3 (6.5%) experienced relapse in the contralateral testis, and 7 (15.2%) had central nervous system (CNS) recurrences. There was a trend toward lower contralateral testis relapse rates in patients who received prophylactic contralateral testicular radiotherapy (RT) compared to those who did not, although this difference did not reach statistical significance (p = 0.054). Similarly, the rate of CNS recurrence did not differ significantly between patients who received CNS prophylaxis (27 with intrathecal methotrexate and 1 with intravenous methotrexate) and those who did not (p = 0.900), nor did the cumulative incidence of CNS relapse (p = 0.54). The 6-month cumulative CNS relapse rates in the prophylaxis and non-prophylaxis groups were 9.5% and 8.5%, respectively, with the median time to CNS relapse among all PT-DLBCL patients being 47.3 months (range: 0.2–106.0).

Among the 7 patients with CNS recurrence, 6 had parenchymal involvement, and 1 had leptomeningeal involvement. The most commonly affected sites were the corpus callosum, basal ganglia, frontal lobe, and parietal lobe. There was no significant difference in CNS relapse rates between patients who received Rituximab and those who did not (p = 1.000). However, patients classified as low-, intermediate-, and high-risk based on CNS-IPI scores (0–1, 2–3, and 4–6, respectively) showed a statistically significant difference in CNS recurrence rates (p = 0.028 **Table 5**).

**Table 5:** CNS relapse in PTLs of different IPI scores.

IPI score, n(%)	CNS recurrence, 7	No CNS recurrence, 39	Total, 46	P value
0-1	0 (0%)	19 (41.3%)	19 (41.3%)	
2-3	5 (10.7%)	12 (26.1%)	17 (37.0%)	
4-6	2 (4.3%)	8 (17.4%)	10 (21.7%)	0.028

#### 3.2.3 Survival analysis by subgroup and prognostic factors for primary testicular DLBCL

**Figure 3:**
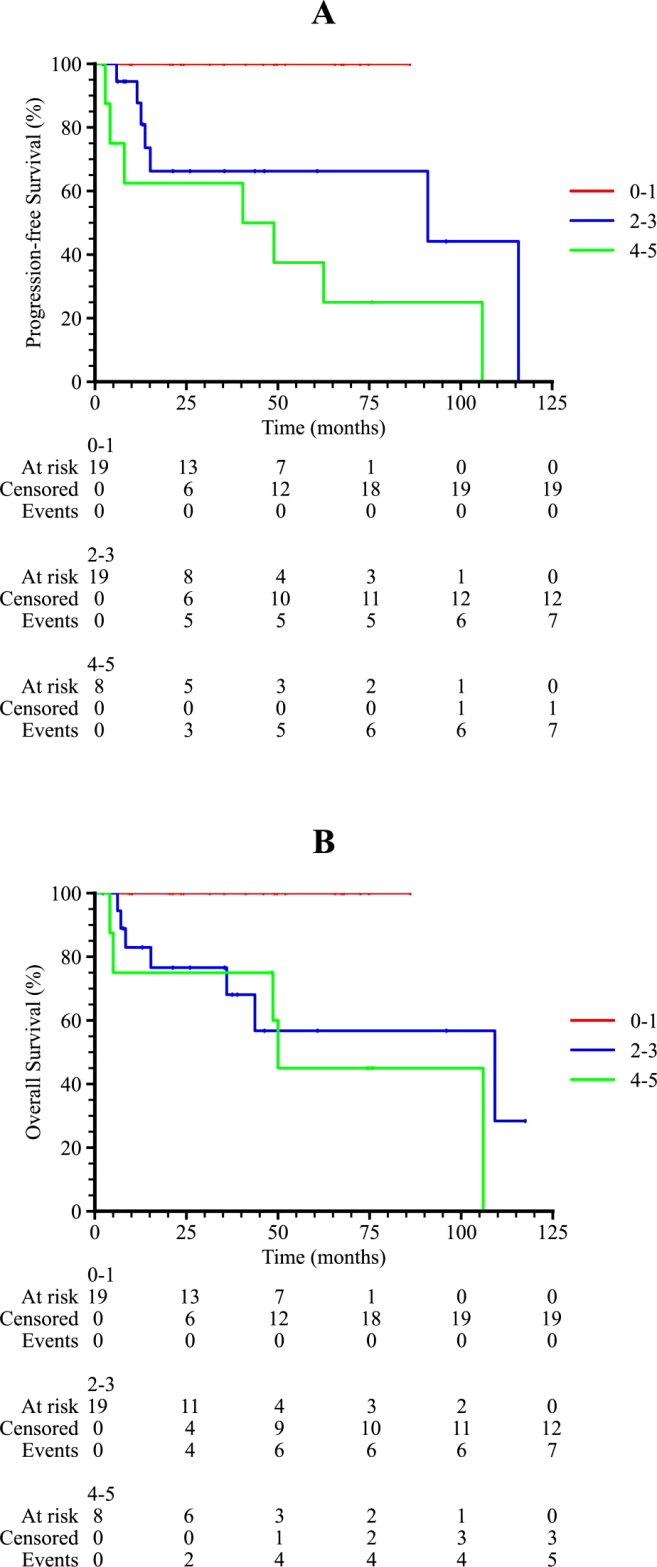
Kaplan-Meier survival curves of PTL patients in different IPI scores. **(A)** The progression-free survival curve among PTL patients in 0-1, 2–3 and 4–5 IPI score. **(B)** The overall survival curve among PTL patients in 0-1, 2–3 and 4–5 IPI score.

**Figure 4:**
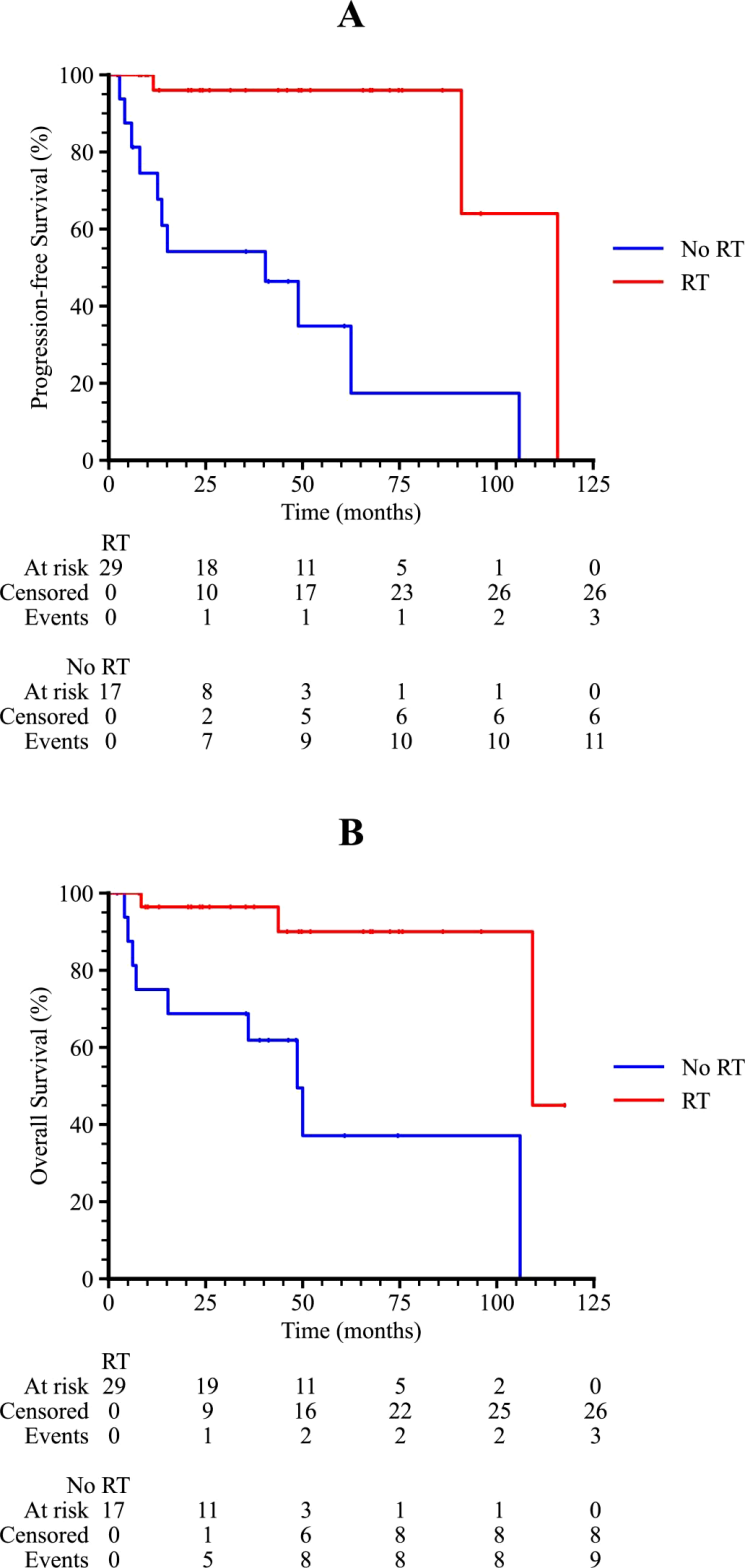
Kaplan-Meier survival curves of PTL patients with or without RT. **(A)** The progression-free survival curve among PTL patients with or without RT. **(B)** The overall survival curve among PTL patients with or without RT.

**Figure 5:**
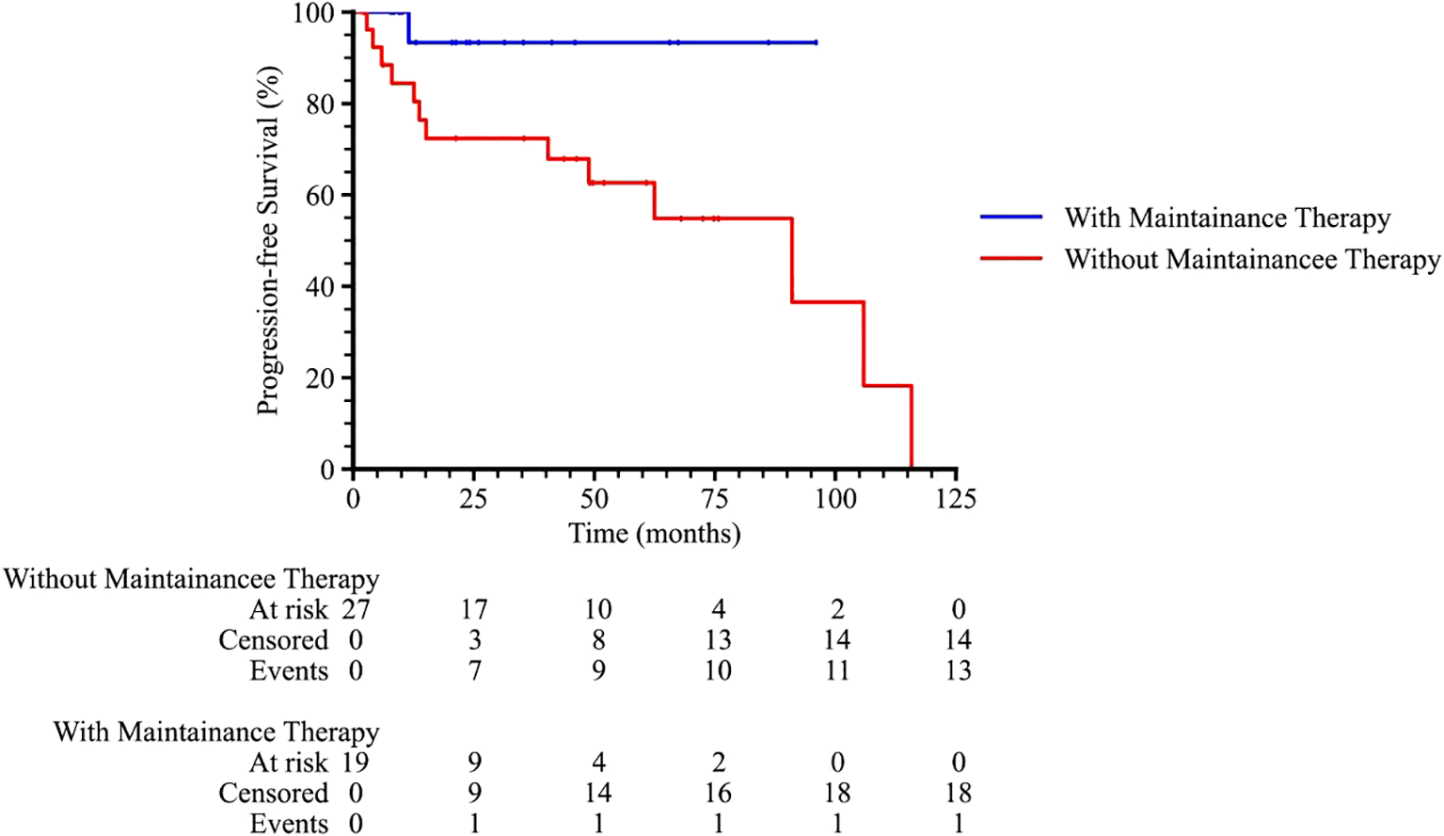
The progression-free survival curve among PTL patients with or without maintenance therapy.

Subgroup analysis revealed that patients with an International Prognostic Index (IPI) score of 2–3 or 4–5 had significantly poorer survival outcomes compared to those with an IPI score of 0–1 (p = 0.001 and 0.011 for PFS and OS, respectively), as shown in **Figure 3**. Patients who received radiotherapy demonstrated improved progression-free survival (PFS) and overall survival (OS) (p = 0.009 and 0.001, respectively, **Figure 4**). The 3-year PFS rate was 93.3% for patients who received maintenance therapy after first-line treatment, compared to 72.4% for those who did not receive maintenance therapy (p = 0.037, **Figure 5**).

Univariate and multivariate Cox regression analyses were performed to evaluate potential prognostic factors in PT-DLBCL patients. In the univariate analysis, age, Ann Arbor stage, IPI score, first-line therapy, and radiotherapy were identified as potential prognostic factors for PFS. For OS, age, IPI score, immunophenotype, first-line therapy, and radiotherapy were identified as possible prognostic factors. Further multivariate analysis confirmed that radiotherapy was an independent prognostic factor for PFS, while the double-expressor phenotype was an independent prognostic factor for OS in PT-DLBCL patients (**Tables 6**, **7**).

**Table 6:** Univariate and multivariate COX analysis in PT-DLBCL patients (PFS).

Variables	Univariate analysis (PFS)		Multivariate analysis (PFS)	
	HR	P value	HR	P value
Age				
	1.09(1.02-1.15)	**0.008**	0.96(0.86-1.07)	0.469
Ann Arbor stage				
I-II III-IV	Ref 12.07(1.54-94.50)	**0.018**	3.45(0.17-70.08)	0.420
COO				
Non-GCB subtype GCB subtype	Ref 0.93(0.24-3.54)	0.916		
Laterality				
Unilateral Bilateral	Ref 1.15(0.28-4.70)	0.848		
IPI score				
	2.26(1.43-3.58)	**<.001**	1.15(0.43-3.08)	0.782
Immunophenotype				
Double-expressor phenotype Not double-expressor phenotype	2.63(0.69-10.13)	0.159		
Double-hit phenotype Not double-hit phenotype	2.05(0.43-9.66)	0.365		
First-line therapy				
No CHOP R-CHOP-like regimens R-EPOCH Others	Ref 0.08(0.01-1.09) 0.12(0.02-0.64) 0.11(0.01-1.32) 0.82(0.10-6.40)	0.058 **0.013** 0.081 0.846	0.05(0.00-1.56) 0.45 (0.06-3.67) 0.17(0.01-4.83) 4.89(0.43-55.96)	0.089 0.457 0.298 0.202
Maintenance therapy				
Yes No	0.15(0.02-1.17) Ref			
Radiotherapy				
Yes No	0.08(0.02-0.35) Ref		0.07(0.01-0.64)	**0.018**
Intrathecal CNS prophylaxis				
Yes No	0.40(0.12-1.32) Ref			

Bold values indicate statistical significance (p < 0.05).

**Table 7:** Univariate and multivariate COX analysis in PT-DLBCL patients (OS).

Variables	Univariate analysis (OS)		Multivariate analysis (OS)	
	HR	P value	HR	P value
Age				
	1.09(1.03-1.15)	**0.004**	1.02(0.92-1.13)	0.750
Ann Arbor stage				
I-II III-IV	Ref 4.06(0.86-19.16)	0.077		
COO				
Non-GCB subtype GCB subtype	Ref 0.70(0.14-3.56)	0.669		
Laterality				
Unilateral Bilateral	Ref 1.63(0.45-5.92)	0.456		
IPI score				
	1.78(1.16-2.73)	**0.008**	0.75(0.40-1.42)	0.378
Immunophenotype				
Double-expressor phenotype Not double-expressor phenotype	6.93(1.95-24.64) Ref	**0.003**	10.99(2.06-58.75)	**0.005**
Double-hit phenotype Not double-hit phenotype	3.43(0.72-16.29) Ref	0.121		
First-line therapy				
No CHOP R-CHOP-like regimens R-EPOCH Others	Ref 0.12(0.01-1.55) 0.11(0.02-0.62) 0.07(0.00-1.15) 1.06(0.15-7.30)	0.104 **0.013** 0.063 0.955	0.13(0.00-4.02) 0.12(0.01-1.25) 0.09(0.00-3.89) 0.53(0.04-6.46)	0.241 0.077 0.208 0.617
Maintenance therapy				
Yes No	0.19(0.02-1.54) Ref	0.120		
Radiotherapy				
Yes No	0.10(0.02-0.49) Ref	**0.004**	0.10(0.01-1.16)	0.065
Intrathecal CNS prophylaxis				
Yes No	0.30(0.08-1.13) Ref	0.075		

Bold values indicate statistical significance (p < 0.05).

## 4 Discussion

Differentiating between PTL and STL remains a significant challenge in clinical practice, impacting clinical staging, treatment planning, and prognostic evaluation. Matthew et al. proposed a stricter definition of PTL, limiting it to testicular lymphomas without lymph node or bone marrow involvement (2). Moreover, Rebecca et al. suggested that phenotypic, genetic, and biological characteristics could further assist in distinguishing these two types (3). PTL, for example, is often associated with the non-GCB subtype and the common mutations of MYD88 L265P and CD79B, although this requires further clinical validation. In our study, we differentiated PTL from systemic lymphoma disease based on clinical symptoms and extranodal involvement. Our findings demonstrate that the two groups exhibited distinct pathological features, treatment responses, and survival outcomes. However, the distinction might be correlated to diverse biological features and treatment strategies, including non-GCB phenotype, MYD88/CD79B mutations, and immune-privileged site involvement between these two groups. Current guidelines recommend that PTL diagnosis be confirmed via biopsy and pathology. Combined treatment strategies, including orchiectomy followed by chemotherapy and radiotherapy, are essential. Orchiectomy not only facilitates an accurate pathological diagnosis but also disrupts the blood-testicular barrier, enhancing the efficacy of chemotherapy. In contrast, for STL patients, diagnosis primarily depends on the intensity of FDG uptake on PET/CT (4, 5). Given the difficulty in interpreting physiologic and pathologic uptake on FDG-PET/CT, we adopted a standardized uptake value (SUVmax) threshold of 5.0 to determine testicular involvement by lymphoma, referencing a previous American study (5). 14 PTLs displayed unfavorable survival outcomes in general, however, one with NKTCL received 6 cycles of DDGP regimen as front-line and then discovered testicular involvement, after 4 cycles of etoposide, PD-1 blockade and chidamide, he received chimeric antigen receptor T-cell (CAR-T) therapy and reached a PFS of 70 months. As systemic chemotherapy has shown limited efficacy in such case, novel therapies such as stem cell transplantation and CAR-T cell therapy should be considered.

Primary testicular diffuse large B-cell lymphoma (PT-DLBCL) is the most common histological subtype of PTL, accounting for 80–90% of cases. It is characterized by a high propensity for relapse in the central nervous system (CNS), contralateral testis, and other extranodal sites (3, 6). However, Chinese real-world data on this rare and aggressive disease remain limited. In our study, we analyzed a cohort of 46 patients with PT-DLBCL, focusing on their clinical features, treatments strategies, and prognosis outcomes. This analysis reflects real-world clinical practice and provides valuable insights into the management of this uncommon lymphoma subtype. The primary symptoms of PT-DLBCL were predominantly unilateral painless swelling, and the most commonly observed phenotype was the non-GCB subtype, which aligns with previous studies (7, 8). Notably, western cohorts have shown greater molecular heterogeneity. Interestingly, half of our patients were at an advanced stage at diagnosis, a higher proportion than previously reported. This may be attributed to frequent misdiagnoses as orchitis, leading to delayed treatment.

Although MYD88 gene mutation testing is not routinely conducted in Chinese clinical practice, our study found an 80% mutation rate, consistent with the 68–82% reported in other Chinese and Asian studies (9). This mutation, frequently co-occurring with CD79B mutations, is a hallmark of the activated B-cell (ABC) or non-GCB subtype, contributing to constitutive NF-κB pathway activation and poorer outcomes. BCL2 and BCL6 mutations, which have also been implicated in extranodal DLBCL prognosis in previous studies, were not assessed in our cohort due to limited access to molecular profiling (10–12). The double- or triple-hit phenotype, commonly involving rearrangements of MYC and BCL-6, was less frequent in PT-DLBCL compared to nodal DLBCL. Similarly, the double-expressor phenotype was also less prevalent in PT-DLBCL (13), while BCL6 rearrangements, double-expressor phenotype, and even double-hit lymphomas involving MYC and BCL6 or BCL2 are reported more frequently in western countries (14).

For PT-DLBCL patients, orchiectomy alone is insufficient; combined treatment modalities are essential for improving outcomes. In the Rituximab era, R-CHOP and R-CHOP-like regimens have been the most commonly used first-line therapies, demonstrating significantly better survival compared to orchiectomy alone. Additionally, Polatuzumab vedotin—a CD79b-targeted antibody–drug conjugate—has shown enhanced efficacy when combined with R-CHP in DLBCL patients, particularly in those with the ABC (non-GCB) subtype. However, its use remains limited in real-world clinical practice in China during the study period. Furthermore, the addition of radiotherapy following chemotherapy has been shown to prolong both progression-free survival (PFS) and overall survival (OS), aligning with the results from IELSG clinical trials (14). Several small molecules and targeted agents, including immunomodulatory drugs (IMiDs) such as lenalidomide and pomalidomide, BTK-i like ibrutinib, zanubrutinib, and orelabrutinib, have been confirmed to exert antitumor effects in B-cell lymphomas by inhibiting BCR signaling and reducing NF-κB pathway activation. These agents have also demonstrated efficacy in crossing the blood-testicular and blood-brain barriers (15–18). In our study, patients who received maintenance therapy with these agents showed a trend toward improved PFS. However, no statistically significant differences in survival were observed based on the specific maintenance regimens used. Interestingly, BTK inhibitors did not display a clear benefit for long-term survival in PT-DLBCL patients, which may be due to their use primarily in the relapsed/refractory (R/R) setting rather than as part of frontline therapy. Only a small number of patients received R-CHOP in combination with agents such as lenalidomide or BTK inhibitors as frontline treatment. In light of recent preclinical and clinical studies highlighting the crucial role of the PD-1/PD-L1 pathway in immune evasion in PT-DLBCL (19, 20), PD-1 blockade was explored in our cohort. One patient achieved a partial response (PR) after receiving a combination of Rituximab and a BTK inhibitor, suggesting that immunotherapy may offer additional therapeutic benefit in select cases.

PT-DLBCL is particularly characterized by a high risk of CNS relapse, occurring in 10%–25% of patients. Although the exact mechanisms underlying CNS recurrence remain unclear, they are thought to involve latent molecular features specific to these immune-privileged sites (21). The CNS-IPI score has been validated as an effective risk stratification model for predicting CNS involvement, consistent with our findings (22, 23). For high-risk patients, implementing appropriate CNS prophylaxis is critical. A phase II study demonstrated that intravenous (IV) high-dose methotrexate (HD-MTX) combined with intrathecal (IT) liposomal cytarabine and R-CHOP was effective in preventing CNS relapse (24). In comparative analyses, IV-directed CNS prophylaxis showed superior survival outcomes over IT methotrexate in high-risk patients (25).However, a real-world study similar to ours found no significant difference in CNS relapse rates between IV and IT regimens (26). In our cohort, due to concerns about the toxicity of IV HD-MTX—especially in elderly patients—only one patient received this regimen. Approximately half of the patients underwent IT MTX prophylaxis, the clinical benefit of which remains controversial. Given that CNS relapses in our patients primarily involved the parenchyma rather than the leptomeninges (6 *vs*. 1), the limited benefit of IT chemotherapy seems reasonable. Altogether, beyond CNS-IPI scoring, genomic biomarkers could also be explored in identifying high-risk patients for CNS recurrence, and CNS-directed chemotherapy should be considered under close health monitoring. However, considering those who are unsuitable for HD-MTX, novel treatment approaches like rational combined therapy with targeted agents such as BTK-i, PD-1 inhibitors and CAR-T cells could be the future direction (27).

Several studies, including retrospective analyses and investigations using large datasets, have explored potential prognostic factors for PT-DLBCL, leading to the development of predictive nomograms (28, 29). However, no unified conclusion has been established thus far. Factors such as age, Ann Arbor stage, laterality, IPI score, and treatment modality have been reported as significant prognostic factors for PT-DLBCL. Interestingly, no obvious difference was observed in intermediate-risk and high-risk PT-DLBCLs when classified by IPI score, perhaps owing to the small number of patients cohort and the insufficient prognostic value of IPI score in PT-DLBCL patients without considering its unique biological characteristics. In our univariate analysis, older patients with advanced-stage disease, high IPI scores, double-expressor phenotypes, orchiectomy alone, and the absence of radiotherapy were associated with poor survival outcomes, consistent with previous reports (30). In multivariate analysis, radiotherapy emerged as an independent prognostic factor for progression-free survival (PFS), while the double-expressor phenotype was an independent factor for overall survival (OS). Given the rarity of this disease and the limited number of cases, our findings warrant further validation in larger, prospective, multi-center studies, and to further optimize prognostic scoring models.

In summary, this retrospective, real-world study included a relatively large cohort of testicular lymphoma patients in China, providing valuable insights into their diagnosis and treatment. Compared with STL, PTL cases tended to demonstrate different pathological subtypes, more favorable clinical responses and survival outcomes, which may reflect earlier disease stage at diagnosis, more localized involvement, and more standardized treatment strategies, based on primary symptoms and extranodal involvement. For PT-DLBCL patients, chemotherapy following orchiectomy, combined with consolidation therapy and radiotherapy, emerged as the recommended therapeutic approach in our study. Future research should focus on optimizing CNS prophylaxis strategies, identifying genomic predictors, and exploring the potential of immunotherapy and targeted therapies for high-risk patients.

## Data Availability

The raw data supporting the conclusions of this article will be made available by the authors, without undue reservation.
